# Ultra-lung-protective ventilation and biotrauma in severe ARDS patients on veno-venous extracorporeal membrane oxygenation: a randomized controlled study

**DOI:** 10.1186/s13054-022-04272-x

**Published:** 2022-12-12

**Authors:** Christophe Guervilly, Théotime Fournier, Juliette Chommeloux, Laurent Arnaud, Camille Pinglis, Karine Baumstarck, Mohamed Boucekine, Sabine Valera, Celine Sanz, Mélanie Adda, Mickaël Bobot, Florence Daviet, Ines Gragueb-Chatti, Jean-Marie Forel, Antoine Roch, Sami Hraiech, Françoise Dignat-George, Matthieu Schmidt, Romaric Lacroix, Laurent Papazian

**Affiliations:** 1 Service de Médecine Intensive Réanimation, Hôpital Nord, Assistance Publique-Hôpitaux de Marseille, Chemin des Bourrely, 13915 Marseille Cedex 20, France; 2 Centre d’Etudes et de Recherches sur les Services de Santé et qualite de vie EA 3279, Aix-Marseille Université, 13005 Marseille, France; 3 Service de Médecine Intensive-Réanimation, Institut de Cardiologie, APHP, Sorbonne, Université Hôpital Pitié- Salpêtrière, Paris, France; 4 INSERM, UMRS_1166-ICAN, Institute of Cardiometabolism and Nutrition, Sorbonne Université, Paris, France; 5 Laboratoire d’Hématologie et de Biologie Vasculaire, Assistance Publique-Hôpitaux de Marseille, Marseille, France; 6 INSERM 1263, Institut National de Recherche Pour l’Agriculture, l’Alimentation et l’Environnement (INRAE), Centre de Recherche en CardioVasculaire et Nutrition (C2VN), Université Aix-Marseille, Marseille, France; 7 Centre de Néphrologie et Transplantation Rénale, AP-HM, Hôpital de la Conception, CHU de la Conception, 13005 Marseille, France; 8 Centre Hospitalier de Bastia, Service de Réanimation, 604 Chemin de Falconaja, 20600 Bastia, France

**Keywords:** Severe ARDS, Veno-venous ECMO, Ultra-lung-protective ventilation, Biotrauma

## Abstract

**Background:**

Ultra-lung-protective ventilation may be useful during veno-venous extracorporeal membrane oxygenation (vv-ECMO) for severe acute respiratory distress syndrome (ARDS) to minimize ventilator-induced lung injury and to facilitate lung recovery. The objective was to compare pulmonary and systemic biotrauma evaluated by numerous biomarkers of inflammation, epithelial, endothelial injuries, and lung repair according to two ventilator strategies on vv-ECMO.

**Methods:**

This is a prospective randomized controlled study. Patients were randomized to receive during 48 h either ultra-lung-protective ventilation combining very low tidal volume (1–2 mL/kg of predicted body weight), low respiratory rate (5–10 cycles per minute), positive expiratory transpulmonary pressure, and 16 h of prone position or lung-protective-ventilation which followed the ECMO arm of the EOLIA trial (control group).

**Results:**

The primary outcome was the alveolar concentrations of interleukin-1-beta, interleukin-6, interleukin-8, surfactant protein D, and blood concentrations of serum advanced glycation end products and angiopoietin-2 48 h after randomization. Enrollment was stopped for futility after the inclusion of 39 patients. Tidal volume, respiratory rate, minute ventilation, plateau pressure, and mechanical power were significantly lower in the ultra-lung-protective group. None of the concentrations of the pre-specified biomarkers differed between the two groups 48 h after randomization. However, a trend to higher 60-day mortality was observed in the ultra-lung-protective group compared to the control group (45 vs 17%, *p* = 0.06).

**Conclusions:**

Despite a significant reduction in the mechanical power, ultra-lung-protective ventilation during 48 h did not reduce biotrauma in patients with vv-ECMO-supported ARDS. The impact of this ventilation strategy on clinical outcomes warrants further investigation.

*Trial registration* Clinical trial registered with www.clinicaltrials.gov (NCT03918603). Registered 17 April 2019.

**Supplementary Information:**

The online version contains supplementary material available at 10.1186/s13054-022-04272-x.

## Background

Veno-venous extracorporeal membrane oxygenation (vv-ECMO) allows decreasing tidal volume (Vt), airway inspiratory pressures, and the respiratory rate (RR) which all individually are able to create or aggravate ventilator-induced lung injuries (VILI) [1]. However, experimental and clinical data suggest that VILI may still occur despite lung-protective ventilation (LP) strategy [2, 3, 4] including low Vt (4–6 mL/kg) of predicted body weight (PBW), low plateau pressure (Pplat) below 28 cm H_2_O and moderate positive end-expiratory pressure (PEEP). Besides, prolonged and repeated prone position (PP) which is recommended in moderate to severe ARDS before vv-ECMO [5, 6] mitigates VILI by promoting a more homogeneous distribution of total lung stress and strain [7] and by reducing biotrauma [8]. Lastly, an individualized strategy of PEEP targeting a positive expiratory transpulmonary pressure (P_*L*_) has been proposed to counteract atelectrauma [9] and to decrease VILI.

On vv-ECMO, gas exchanges are mainly ensured by the ECMO membrane. Prospective randomized studies have suggested a possible interest to target positive expiratory P_*L*_ by PEEP setting [10] and using an ultra-lung-protective (ULP) ventilation with both reduced Vt and driving pressure [11, 12]. Of note, retrospective aggregated data suggest a potential benefit of continuation or initiation of PP in vv-ECMO patients [13, 14].

However, there are no formal guidelines on how to ventilate vv-ECMO patients to optimize lung rest. In the absence of strong evidence, an approach based on the ventilation protocol of the ECMO arm of the EOLIA trial is generally applied [15]. This strategy combined Pplat reduction ≤ 24 cm H_2_O, driving pressure ≤ 14 cm H_2_O while PEEP is maintained ≥ 10 cm H_2_O [15]. Of note, in the EOLIA trial, the RR which is one of the main components of mechanical power was not markedly reduced and maintained close to 23 cycles/min after ECMO onset [16]. Since this trial, physiological studies have suggested that decreasing further the mechanical power of mechanical ventilation during ECMO might be beneficial [3, 12, 17].

Herein, we hypothesize that compared with a lung-protective (LP) strategy as performed in the EOLIA trial, an ultra-lung-protective (ULP) ventilation strategy including a very reduced Vt (1–2 mL/kg of predicted body weight), a low RR (5–10 cycles/min), a positive expiratory P_*L*_ and the use of PP could reduce the biotrauma and therefore enhance VILI prevention.

## Methods

### Study setting

We performed a prospective single-blinded randomized controlled study fulfilling the 2010 CONSORT guidelines (Additional file 1) in two tertiary university ECMO centers in Marseille (Hôpital Nord) and Paris (La Pitié-Salpétrière) in France. We included intubated and mechanically ventilated adults with severe ARDS [18] receiving vv-ECMO for less than 24 h. The main exclusion criteria were > 24 h of vv-ECMO support, contraindication for prone positioning, contraindication for esophageal catheter, and current treatment with steroids (> 0.5 mg/kg/day of equivalent methylprednisolone) (ESM). Each patient or surrogate had to give a written informed consent. The study was registered in the database ClinicalTrial.gov on April 17th, 2019 (NCT03918603).

### Study design

**Fig. 1:**
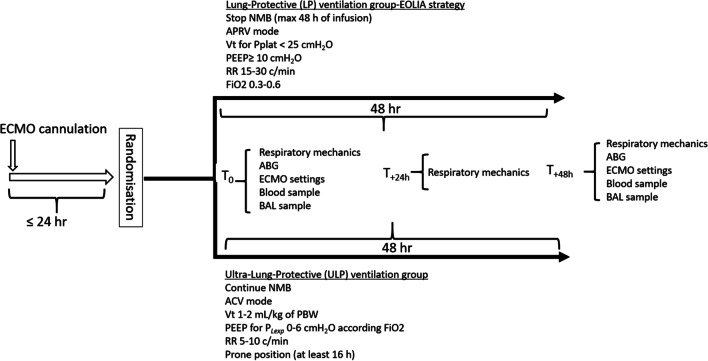
Study design. *ECMO* extracorporeal membrane oxygenation, *NMB* neuromuscular blocker, *APRV* airway pressure release ventilation, *Vt* tidal volume, *PEEP* positive end-expiratory pressure, *RR* respiratory rate, *ACV* assist controlled ventilation, *BAL* broncho-alveolar lavage, *ABG* arterial blood gas

The study design is presented in Fig. 1. Patients were randomized at a 1:1 ratio either to the LP or to the ULP group using a Web-based system. Randomization was stratified according to the center (blocks of 4).

### Experimental arm (i.e., ULP group)

Neuromuscular blockers (NMB) were continuously infused for 48 h while mechanical ventilation included a Vt of 1–2 mL/kg of predicted body weight (PBW) at a RR of 5–10 cycles /min in the volume-controlled mode. PEEP was settled to target a positive P_*L*_ obtained through an esophageal balloon catheter (Nutrivent™, Sidam, Mirandola, Italy) inflated with a minimal, non-stress volume (3–4 ml) of air (see the online data supplement for other details regarding esophageal pressure measurement method). Besides, patients had to be proned for at least 16 h during this 48-h study period. Of note, PEEP was titrated to obtain positive expiratory transpulmonary pressure only in the supine position.

### Control arm (i.e., LP group)

First, patients were maintained in the supine position for 48 h. Mechanical ventilation followed the ECMO arm of the EOLIA trial with combined early interruption of NMB infusion, Pplat < 25 cm H_2_O, PEEP ≥ 10 cm H_2_O, RR of 15–30 cycles/min. An airway pressure release ventilation (APRV) mode was encouraged with time low at least twofold longer than time high.

In both arms, mechanical ventilation was left at the physician’s discretion after 48 h as PP and NMB continuation.

### Measurements

In both arms, broncho-alveolar lavage (BAL) and blood sampling were performed at baseline (T_0_) and repeated 48 h after randomization (T_+48 h_). BAL and blood samples were rapidly centrifuged at room temperature, respectively, at 300× g for 5 min and at 2500× g for 15 min and stored at − 80 °C until analysis was performed by an independent laboratory blinded to the randomization arm. The full list of biomarkers analyzed in the BAL and plasma with their specificity and assay details is provided in the online supplement (Additional file 2: Table S1). Besides, respiratory mechanics, arterial blood gas analyses, ECMO settings, hemodynamics, and complications (barotrauma (pneumothorax, pneumomediastinum), right ventricular dysfunction, pressure sores, and tracheal tube obstruction) were daily recorded from baseline (T_0_) to day 6.

### Primary and secondary outcomes

The main objective was to demonstrate that a ULP ventilation applied during 48 h was associated with a decrease in pre-specified biomarkers in BAL and blood. The primary outcomes were concentrations of interleukin-1-beta (IL-1-beta), interleukin-6 (IL-6), interleukin-8 (IL-8), and surfactant protein D (SP-D) in broncho-alveolar lavage (BAL) and blood concentrations of serum advanced glycation end products (sRAGE) and angiopoietin-2 (Ang-2) 48 h after randomization (T_+48 h_).

Secondary outcomes were ECMO weaning rate, 60-day, ICU, and hospital mortality rates, and biomarkers concentrations in BAL and blood not specified as the primary outcome on T + 48 h. Delta concentrations (T + 48 h minus T0) were also computed and compared between groups. Prone position-related complications (pressure sores and tracheal tube obstruction), occurrences of barotrauma (pneumothorax, pneumomediastinum), and right ventricular dysfunction were also compared.

### Sample size and statistical analysis

We anticipated a difference of 8 ± 10 pg/mL of IL-1-beta in BAL on T_+48 h_ between the ULP group and the LP group [18, 19]. The calculated sample size was 52 patients with a power of 80% and risk α of 5%. We planned to include 60 patients to avoid missing data due to early deaths (< 48 h after inclusion) and technical issues due to the BAL sample. We planned an interim analysis after the inclusion of two-thirds of the patients. All analyses were performed with the intention to treat. Additional details are available in the electronic supplementary material.

## Results

### Patients

In total, 310 patients with severe ARDS on vv-ECMO were screened for eligibility (Additional file 2: Fig. S1). In the interim analysis, we randomized 39 patients, 20 to the ULP group and 19 to the LP group. The first patient was enrolled on July 1, 2019, and the last on April 23, 2021. The last patient’s outcome was obtained on June 22, 2021. For understandable logistical reasons, enrollment was interrupted during the first wave of COVID-19 pandemic in France. Inclusions were stopped for futility after the interim analysis. Consent to use primary outcome data were withdrawn for one patient assigned to the LP group. Thus, thirty-eight patients (20 in the ULP group and 18 in the LP) were included in the primary analysis. Pre-ECMO characteristics were well balanced between the two groups (Table 1) except for a significantly higher Vt in the LP group. Half of the patients in each group had COVID-19-related ARDS. Time from vv-ECMO cannulation to randomization was 16 ± 7 h in the ULP group and 16 ± 6 h in the LP group (*p* = 0.87).

**Table 1:** Pre-ECMO and baseline (T_0_) characteristics of patients

Variable	Ultra-lung-protective group		Lung-protective group	
	*N*	n (%)*	*N*	n (%)*
Age, years, median (IQR)	20	56 (41–65)	18	57 (48–61)
Male sex,	20	13 (65)	18	12 (67)
Body mass index, kg/m^2^, median (IQR)	20	28 (25–33)	18	31 (27–36)
SAPS 2 at admission, median (IQR)	20	41 (35–49)	18	45 (32–56)
SOFA score at inclusion, median (IQR)	20	8 (4–11)	18	8 (5–9)
Cause of ARDS				
COVID-19	20	10 (50)	18	9 (50)
Non COVID-19 CAP	20	7 (35)	18	5 (28)
Aspiration	20	2 (10)	18	3 (17)
Lung contusion	20	1 (5)	18	0 (0)
Extra-pulmonary sepsis	20	0	18	1 (5)
Comorbidity				
Ischemic cardiomyopathy	20	1 (5)	18	0 (0)
Diabetes	20	5 (25)	18	2 (11)
Chronic renal insufficiency	20	0 (0)	18	1 (5)
Chronic respiratory disease	20	3 (15)	18	2 (11)
Immunocompromised	20	1 (5)	18	1 (5)
Before ECMO, median (IQR)				
Duration of mechanical ventilation, days	20	8 (6–12)	18	4 (1–12)
Vasopressors	20	10 (50)	18	8 (44)
Renal replacement therapy	20	0 (0)	18	1 (5)
Rescue therapy pre-ECMO				
Any	20	20 (100)	18	18 (100)
Continuous infusion of NMB	20	18 (90)	18	17 (94)
Prone position	20	17 (85)	18	16 (89)
Inhaled nitric oxide	20	10 (50)	18	10 (55)
Almitrine infusion	20	2 (10)	18	2 (11)
Respiratory mechanics at ECMO cannulation, median (IQR)				
FiO_2_, %	19	100 (100–100)	18	100 (100–100)
PEEP, cm H_2_O	16	14 (10–15)	15	14 (12–15)
Tidal volume, mL/PBW	15	5.6 (5.4–6.1)	15	6.5 (5.9–6.9)
Respiratory rate, breaths/min	14	30 (27–30)	15	26 (24–30)
Minute ventilation, L/min	14	11.6 (9.7–13.1)	13	10.1 (9.3–12.1)
Plateau pressure, cm H_2_O	14	30 (28–32)	12	31 (25–33)
Peak pressure, cmH_2_O	12	38 (37–45)	6	39 (37–41)
Driving pressure, cm H_2_O	14	16 (14–19)	12	17 (13–20)
RS compliance, mL/cm H_2_O	14	21 (19–29)	12	24 (18–34)
Mechanical power, J/min	11	35 (25–43)	6	28 (25–33)
Ventilatory ratio	12	2.6 (2.4–3.1)	10	2.5 (1.93.5)
Last blood gas values pre-ECMO, median (IQR)				
pH	14	7.27 (7.21–7.33)	14	7.24 (7.18–7.37)
PaO_2_:FiO2, mm Hg	18	74 (55–106)	17	83 (72–94)
PaCO_2_, mm Hg	11	68 (55–72)	11	59 (50–71)
Lactates, mmol/L	14	1.8 (1.0–2.5)	13	1.5 (1.1–2.0)
ECMO configuration				
Femoral–jugular	20	14 (70)	18	16 (89)
Femoral–femoral	20	6 (30)	18	2 (11)
Respiratory mechanics and ventilator settings at baseline (T_0_)				
FiO_2_, %	16	100 (77–100)	17	100 (50–100)
PEEP, cm H_2_O	18	13 (12–15)	16	14 (12–15)
Tidal volume, mL/PBW	18	2.6 (1.9–3.8)	18	3.5 (2.8–4.2)
Respiratory rate, breaths/min	18	13 (10–15)	18	15 (11–21)
Minute ventilation, L/min	17	2.2 (1.4–3.0)	15	3.1 (2.2–6.1)
Plateau pressure, cm H_2_O	16	24 (21–25)	15	25 (24–27)
Peak airway pressure, cmH_2_O	14	28 (24–30)	14	29 (24–36)
Driving pressure, cm H_2_O	18	10 (7–15)	14	11 (9–15)
RS compliance, mL/cm H_2_O	18	17 (14–21)	14	21 (13–35)
Mechanical power, J/min	13	2.7 (1.7–3.9)	12	3.9 (2.9–5.6)
ECMO settings				
ECMO blood flow L/min	18	4 (3.7–5.0)	18	4.6 (3.9–5.2)
Sweep gas flow, L/min	18	4 (3–5)	18	3.5 (3–5)
Membrane FmO_2_, %	20	100 (100–100)	18	100 (100–100)
Arterial blood gas				
pH	17	7.40 (7.36–7.44)	18	7.40 (7.34–7.45)
PaO_2_, mmHg	17	82 (69–115)	18	97 (73–118)
PaCO_2_, mmHg	17	49 (40–56)	18	43 (37–51)
Plasma bicarbonate, mmol/L	17	30 (25–33)	18	27 (22–31)
SaO_2_, %	17	98 (96–99)	18	99 (97–99)
Lactates, mmol/L	16	1.7 (1.2–3.2)	18	1.5 (1.0–3.1)

*IQR* interquartile range, *SAPS 2* simplified acute physiology score, *SOFA* sequential organ failure assessment score, *ARDS* acute respiratory distress syndrome, *COVID-19* coronavirus disease 2019, *CAP* community acquired pneumonia, *ECMO* extracorporeal membrane oxygenation, *NMB* neuromuscular blockers, *PEEP* positive end-expiratory pressure, *PBW* predicted body weight, *RS* respiratory system, *PaO*_*2*_*:FiO*_*2*_ the ratio of the partial pressure of arterial oxygen to the fraction of inspired oxygen, *PaO*_*2*_ partial pressure of arterial oxygen, *PaCO*_*2*_ partial pressure of arterial carbon dioxide, *FmO2* fraction of membrane oxygen

Driving pressure was defined as plateau pressure minus positive end-expiratory pressure. Static compliance was defined as tidal volume divided by driving pressure. Mechanical power (MP) was calculated as follows: MP = 0.098 × tidal volume x respiratory rate x (peak airway pressure − ½ × driving pressure). Ventilatory ratio was defined as [minute ventilation (ml/min) × PaCO2 (mmHg)] / (predicted body weight × 100 × 37.5). *Unless otherwise indicated

### Respiratory mechanics, ventilator, and ECMO settings in each group

#### During the first 48 h of the study (protocol timeline)

Respiratory mechanics, ventilator, and ECMO settings at baseline (T0) are displayed in Table 1 and Additional file 2: Fig. S2. At baseline (T_0_), tidal volume, minute ventilation, and mechanical power were significantly lower in the ULP group. Tidal volume, respiratory rate, minute ventilation, plateau pressure, and mechanical power were significantly lower in the ULP group on T_+24 h_ and T_+48 h_ (Table 2 and Additional file 2: Fig. S2). Mechanical power was considerably reduced on T_+48 h_ in both groups (by a factor of 10 in the ULP group and 6 in the LP group as compared with pre-ECMO). PEEP, driving pressure, and respiratory system compliance were not different between groups from T_0_ to T_+48 h_ (Table 2 and Additional file 2: Fig. S2). Expiratory transpulmonary pressure was maintained positive on day 1 and day 2 in the ULP group, + 3 (2–5) and + 2 (1–3) cm H_2_O.

**Table 2:** Ventilator and ECMO settings, respiratory mechanics, and gas exchanges from T0 to H + 48 h

Variable	T_0_			H_+24 h_			H_+48 h_		
	Ultra-lung-protective group	Lung-protective group	*p* value	Ultra-lung-protective group	Lung-protective group	*p* value	Ultra-lung-protective group	Lung-protective group	*p* value
Ventilatory mode ACV/APRV/PSV, n (%)	19 (95)/1 (5)/0 (0)	13 (72)/5 (28)/0 (0)	0.055	19 (95)/1 (5)/0 (0)	13 (72)/5 (28)/0 (0)	0.055	17 (85)/3 (20)/ 0 (0)	12 (67)/ 6 (33)/ 0 (0)	0.18
No. of patients	20	18		20	18		20	18	
Tidal volume (mL/PBW)	2.6 (1.9–3.8)	3.5 (2.8–4.2)	0.04	1.8 (1.7–2.0)	2.9 (2.4–3.9)	< 0.001	1.9 (1.6–2.0)	3.3 (2.4–3.8)	0.001
No. of patients	18	18		19	18		20	18	
Respiratory rate (breaths/min)	13 (10–15)	15 (11–21)	0.34	10 (10–13)	15 (12–20)	0.003	10 (10–10)	15 (11–22)	0.001
No. of patients	18	18		20	18		20	18	
PEEP (cmH_2_O)	13 (11–14)	14 (12–15)	0.38	13 (10–16)	12 (12–15)	0.73	12 (10–15)	14 (12–14)	0.23
No. of patients	17	16		19	15		18	15	
Plateau pressure (cmH_2_O)	24 (21–25)	25 (24–27)	0.07	24 (20–24)	24 (23–26)	0.04	21 (18–23)	24 (22–26)	0.01
No. of patients	17	15		19	15		19	15	
Minute ventilation (L/min)	2.2 (1.4–3.0)	3.1 (2.2–6.1)	0.03	1.3 (1.1–1.4)	2.8 (2.1–5.1)	< 0.001	1.3 (1.1–1.5)	2.8 (2.1–5.1)	0.001
No. of patients	18	18		19	18		20	18	
Driving pressure (cmH_2_O)	10 (7–15)	11 (9–15)	0.59	10 (7–15)	10 (8–13)	0.25	9 (6–11)	10 (8–14)	0.21
No. of patients	17	14		19	13		18	13	
Respiratory system compliance (mL/cmH_2_O)	17 (14–21)	21 (13–35)	0.2	13 (10–19)	19 (10–24)	0.25	15 (11–22)	20 (11–32)	0.46
No. of patients	17	14		19	13		18	13	
P_*L. exp*_ (cmH_2_O)	3 (1–4)	6 (4–6)	0.07	3 (2–5)	− 2 (− 4 to + 5)	0.2	2 (1–3)	1.5 (− 2 to + 5)	1
No. of patients	7	3		11	8		8	8	
Mechanical power (J/min)	2.7 (1.7–3.9)	3.9 (2.9–5.6)	0.04	2.5 (2.0–2.6)	5.3 (3.7–6.5)	< 0.001	2.4 (2.2–3.2)	5.4 (3.3–6.5)	0.003
No. of patients	12	12		13	13		14	13	
ECMO blood flow (L/min)	4 (3.7–5.0)	4.6 (3.9–5.2)	0.31	4.5 (3.9–4.8)	4.3 (3.6–5.0)	0.63	4.4 (4.1–5.0)	4.0 (3.5–5.1)	0.18
No. of patients	18	18		20	18		20	18	
Sweep gas flow (L/min)	4 (3–5)	3.5 (3–5)	0.91	5 (4–7)	4 (3.5–6)	0.34	6 (5–7)	4.5 (3.5–6)	0.04
No. of patients	18	18		18	18		19	18	
Membrane FmO_2_ (%)	100 (100–100)	100 (100–100)	1	100 (100–100)	100 (100–100)	1	100 (100–100)	100 (100–100)	1
No. of patients	20	18		20	18		20	18	
pH	7.40 (7.36–7.44)	7.40 (7.34–7.45)	0.88	7.41 (7.37–7.45)	7.41 (7.36–7.45)	0.72	7.37 (7.35–7.45)	7.41 (7.34–7.44)	0.85
No. of patients	17	18		20	18		20	18	
PaO_2_ (mmHg)	82 (69–115)	97 (73–118)	0.71	82 (68–98)	81 (69–104)	0.88	75 (69–83)	81 (68–92)	0.48
No. of patients	17	18		20	18		20	18	
PaCO_2_ (mmHg)	49 (40–56)	43 (37–51)	0.23	47 (45–53)	45 (41–53)	0.41	48 (44–54)	47 (43–57)	0.67
No. of patients	17	18		20	18		20	18	

Values are expressed as median (interquartile range) or number (%)

*ACV* assisted controlled ventilation, *APRV* airway pressure release ventilation, *PSV* pressure support ventilation, *PBW* predicted body weight, *PEEP* positive end-expiratory pressure, *P*_*L. exp*_ expiratory transpulmonary pressure

Driving pressure was defined as plateau pressure minus positive end-expiratory pressure. PaO_2_ = partial pressure of arterial oxygen; PaCO_2_ = partial pressure of arterial carbon dioxide. FmO2 = fraction of membrane oxygen. Static compliance was defined as tidal volume divided by driving pressure. Expiratory transpulmonary pressure (P_*L. exp*_) was calculated as total PEEP minus expiratory esophageal pressure. Mechanical power (MP) was calculated as follows: MP = 0.098 × tidal volume x respiratory rate x (peak airway pressure − ½ × driving pressure)

From T_0_ to T_+48 h_, all patients of the ULP group were turned in PP at least once, and 5 patients (25%) had two PP sessions. Time from vv-ECMO cannulation to first prone positioning in the ULP group was 19 ± 8 h. The median duration of PP by session was 17 h for the first PP session and 16 h for the second PP session. In the LP group, 6 patients (33%) were switched from assisted controlled ventilation (ACV) to APRV mode. ECMO blood flow and membrane FmO_2_ were not different between groups from H_0_ to H + 48 (Table 2). Sweep gas flow was higher in the ULP group at H + 48. Gas exchanges were not different between groups from H_0_ to H + 48 (Table 2).

#### From day 3 to day 6

Tidal volume, plateau pressure, PEEP, driving pressure, respiratory system compliance, and mechanical power were not different between the two groups from day 3 to day 6 (Additional file 2: Fig. S2). ECMO blood flow, sweep gas flow and FmO_2_ did not differ between groups from day 3 to day 6. From day 3, PP was realized at the discretion of the medical team in charged and performed in both groups. Notably, its use increased up to 33% in the LP group and decreased to 5% in the ULP group at day 6.

### Biomarkers and surrogates of biotrauma

The specificity characteristics and the sample site of each biomarker are listed in Table S.1. At baseline, BAL and blood biomarkers were well balanced between ULP and LP groups (Table S.2).

#### Primary outcome

**Fig. 2:**
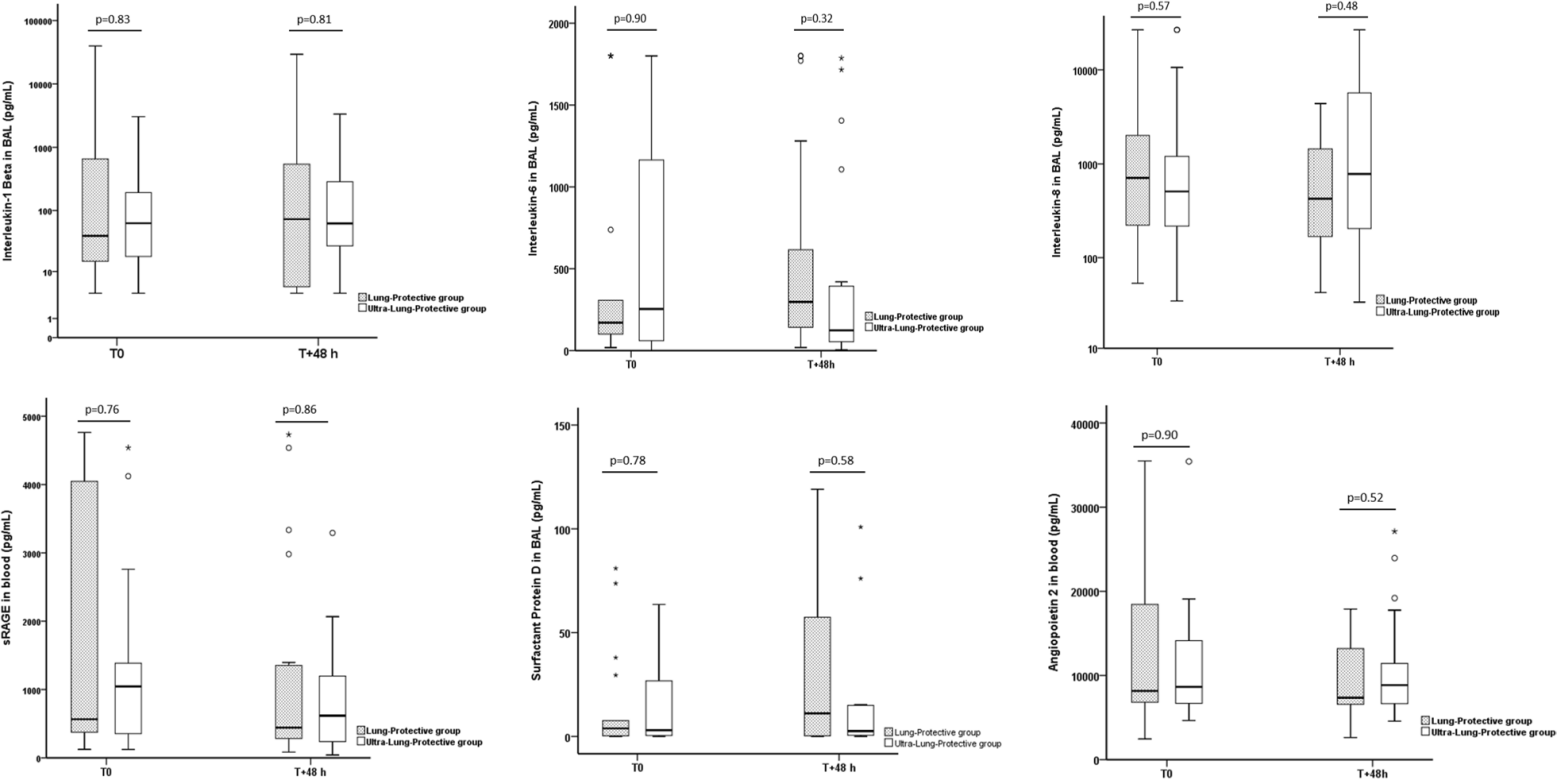
Comparisons of alveolar interleukin-1-beta, interleukin-6, interleukin-8, surfactant protein D and blood concentrations of serum advanced glycation end products and angiopoietin-2 at baseline (T0) and T + 48 h between lung-protective group and ultra-lung-protective group. *sRAGE* serum advanced glycation end products. The empty circles represent the outliers and the black stars represent the extreme values

At T_+48 h_, concentrations of alveolar IL-1-beta were not different between the two groups (Fig. 2 and Table 3). BAL concentrations of IL-6, IL-8, SP-D and blood concentrations of sRAGE were not different between ULP and LP groups at T_+48 h_ (Fig. 2 and Table 3).

**Table 3:** Biomarkers concentrations at T_+48 h_

Biomarker	Primary outcome	Sample	Technic of measure, unit	Ultra-lung-protective group		Lung-protective group		*p* value
				*N*		*N*		
Interleukin-1 Beta	Yes	BAL	ELISA, pg/mL	20	73 (5–548)	18	71 (28–349)	0.81
Interleukin-1 Beta	No	Serum	ELISA, pg/mL	20	0.7 (0.3–0.8)	18	0.4 (0.3–1)	0.36
sRAGE	No	BAL	ELISA, pg/mL	20	114 (50–2002)	18	715 (395–2736)	0.27
sRAGE	Yes	Plasma	ELISA, pg/mL	20	831 ± 796	18	1249 ± 1541	0.86
Angiopoietin 2	Yes	Plasma	Luminex, pg/mL	20	10,735 ± 6356	18	8753 ± 4084	0.52
TNF receptor 1	No	BAL	ELISA, pg/mL	20	815 ± 593	18	792 ± 418	0.90
TNF receptor 1	No	Plasma	ELISA, pg/mL	20	5756 ± 3308	18	6425 ± 5106	0.86
TNF-alpha	No	BAL	Luminex, pg/mL	20	5.6 (1.6–16.8)	18	6.9 (1.9–32.6)	0.86
TNF-alpha	No	Serum	Luminex, pg/mL	20	32 ± 28	18	36 ± 30	0.70
Interleukin-1ra	No	BAL	Luminex, pg/mL	20	4.9 (1–16.3)	18	3.2 (2.1–7.9)	0.94
Interleukin-6	Yes	BAL	Luminex, pg/mL	20	153 (52–935)	18	256 (136–599)	0.32
Interleukin-8	No	Serum	Luminex, pg/mL	20	47 (20–69)	18	52 (27–204)	0.55
Interleukin-8	Yes	BAL	Luminex, pg/mL	20	563 (165–8963)	18	432 (156–1525)	0.48
Interleukin-10	No	Serum	Luminex, pg/mL	20	38 ± 45	18	50 ± 67	0.52
Interleukin-10	No	BAL	Luminex, pg/mL	20	9.4 (2.6–32.5)	18	7.1 (2.9–16.9)	0.70
IP10	No	BAL	Luminex, pg/mL	19	214 (45–682)	18	538 (144–1534)	0.33
Procollagen 3	No	BAL	ELISA, ng/mL	20	2.6 (1–16.3)	18	2.4 (1–13.4)	0.92
MMP-9	No	BAL	ELISA, ng/mL	20	650 ± 737	18	683 ± 559	0.44
Surfactant protein D	Yes	BAL	ELISA, ng/mL	20	2.5 (0.5–15.2)	18	11.1 (0.3–66.6)	0.58
Clara cell protein 16	No	BAL	ELISA, ng/mL	20	5635 ± 6617	18	4489 ± 5638	0.70
Clara cell protein 16	No	Plasma	ELISA, ng/mL	20	56 ± 37	18	58 ± 39	0.93
VEGF	No	Plasma	ELISA, pg/mL	20	61 (20–78)	18	60 (26–194)	0.53
vWF antigen	No	BAL	ELISA, mU/mL	20	1.8 (0.6–19.1)	18	9.4 (0.4–25.1)	0.59

Values are expressed as median (interquartile range) or mean ± standard deviation

*BAL* broncho-alveolar lavage, *ELISA* enzyme-linked immune assay, *sRAGE* serum advanced glycation end products, *TNF* tumor necrosis factor, *Interleukin-1ra* interleukin-1 receptor antagonist, *IP10* Interferon gamma-induced protein 10, *MMP-9* matrix metalloproteinase 9, *VEGF* vascular endothelial growth factor, *vWF* von Willebrand factor

#### Secondary outcomes

BAL and blood concentrations of the other biomarkers investigated were not different between ULP and LP groups at T_+48 h_ (Table 3). Values of delta (T_+48 h_ minus T_0_) of biomarkers concentration are provided in Additional file 2: Table S3. Blood concentrations of tumor necrosis factor α (TNFα), interleukin-8 (IL-8), and vascular endothelial growth factor (VEGF) decreased from baseline to T_+48 h_ in the ULP group but not in the LP group (*p* = 0.02, *p* = 0.04 and *p* = 0.001, respectively) (Additional file 2: Fig. S3). We performed a subgroup analysis of biomarkers at T_+48 h_ restricted to the COVID-19 patients. We did not find any differences in biomarkers concentrations at T_+48 h_ between ULP and LP groups.

### Clinical outcomes and safety issues

Clinical outcomes are presented in Table S.4. Unadjusted 60-day mortality rate (as well as unadjusted hospital mortality rate) was 45% in the ULP group and 17% in the LP group (*p* = 0.06). Sixty-day and hospital mortality rates of the 191 eligible but not randomized patients were 37% and 38%, respectively. Occurrences of barotrauma (pneumothorax, pneumomediastinum), right ventricular dysfunction, pressure sores, and tracheal tube obstruction were not different during the 48 h of the ventilation protocol (respectively, 5% in the ULP group and 5.6% in the LP group, *p* = 0.94, 10% in the ULP group and 5.6% in the LP group, *p* = 0.61). No patient had presented tracheal tube obstruction. At baseline, procollagen 3 in BAL was associated with 60-day and hospital mortality (*p* = 0.025). At 48 h, interleukin-6, interleukin-10 in BAL and blood sRAGE were associated with 60-day and hospital mortality (*p* = 0.025, *p* = 0.013 and *p* = 0.006, respectively).

## Discussion

In this randomized single-blinded controlled study evaluating during 48 h two strategies of ventilation during vv-ECMO in severe ARDS patients, we did not observe a major impact on biotrauma of an ultra-lung-protective multimodal strategy associating very low tidal volume, low respiratory rate, positive expiratory transpulmonary pressure and intermittent prone position as compared with the lung-protective strategy of the early ECMO arm of the EOLIA trial. The pathophysiological rationale to use a ULP approach was attractive, but this large exploration of the biotrauma did not highlight any relevant beneficial effect of the investigated strategy.

We reported an unadjusted nonsignificant difference of 60-day and hospital mortality rates of 45% in the ultra-lung-protective group and 17% in the lung-protective group. However, both the study design and power did not allow any conclusion regarding the impact of the ULP strategy regarding clinical outcomes. Further studies should explore this aspect.

No large, prospective clinical trials comparing different ventilation strategies during vv-ECMO have been published. Therefore, no strong guidelines have been established. Available data, notably from the EOLIA trial, might offer insights into what might be considered current best practices.

Unresolved issues regarding the ventilator settings (notably PEEP and respiratory rate), role of adjunctive therapies (NMB, prone position), or facilitation of spontaneous breathing during ECMO are suggested as areas for future research [20].

Therefore, the present study can provide even very partial insights into the questions raised. How far can be decreased the intensity of mechanical ventilation during vv-ECMO and the potential risks of ultra-lung-protective invasive mechanical ventilation have been recently discussed [21].

We also provide some data on the feasibility and safety of multimodal ultra-lung-protective ventilation strategy which minimizes mechanical power transmitted to a level very close to quasi-apneic ventilation (< 3 J/min) [22].

Previous studies have investigated different mechanical ventilation strategies on biotrauma during ECMO.

Araos et al., in a pig model of ARDS, found that near-apneic ventilation (PEEP, 10 cm H_2_O; driving pressure, 10 cm H_2_O; respiratory rate, 5 bpm) compared with conventional protective ventilation (PEEP, 10 cm H_2_O; VT, 6 ml/kg; respiratory rate, 20 bpm) decreased histologic lung injury, matrix metalloproteinase activity, and prevented the expression of fibro-proliferation [3].

Del Sorbo et al. performed a crossover study in severe ARDS patients on vv-ECMO. Patients were randomly assigned to receive either pressure-controlled ventilation 20 cm H_2_O for 2 h or continuous positive airway pressure for 2 h [12]. The authors found a linear relationship between the change in driving pressure and the plasma concentration of biomarkers of epithelial injury, IL-6, sRAGE, IL-1ra, TNF-alpha, SP-D, and IL-10, suggesting that biotrauma may occur even with very low tidal volume (≈ 2.5 mL/kg of PBW). Of note, this relationship was not observed for other inflammatory biomarkers such as IFN gamma, IL-1 beta, and IL-8.

Different results have been published by Rozencwajg et al. [11]. In this crossover study performed on 16 ARDS patients on vv-ECMO, they did not find change in plasma and broncho-alveolar lavage of sRAGE, plasma IL-6, and monocyte chemotactic protein-1 with different mechanical ventilation strategies with a range of driving pressure between 4 and 19 cmH_2_O resulting in inspiratory pressures between 17 and 24 cmH_2_O.

Last, Amado‑Rodríguez et al. did not find differences in numerous inflammatory biomarkers sampled by BAL between ventilation protocol with Vt of 3 or 6 mL/kg of PBW during 24 h in patients supported by veno-arterial ECMO for cardiogenic shock [23].

These discrepancies need cautious interpretations. First, differences between biomarkers were mainly observed when tidal volume or driving pressure was considerably reduced by the ventilation protocol [3, 12]. Second, when a lung-protective strategy was also applied in the control group, the intervention aiming to minimize the tidal volume and/or driving pressure did not demonstrate a significant reduction in biotrauma [11, 23]. Finally, the design (cross-over vs randomized study), the duration of the mechanical ventilation protocol (range 9–36 h) and the studied models (human vs pig) were different across the studies.

*Princeps* studies that have demonstrated differences in alveolar and systemic biotrauma, used high differences in Vt (mean difference of 6 mL/kg of PBW) and PEEP (mean difference of 10 cmH_2_O) between the control group (high Vt, low PEEP) and the lung-protective group (low Vt, high PEEP) [24, 25]. The effect on biotrauma could be observed as soon as 1–2 h after changes in ventilatory strategy.

In a randomized controlled study, Bein et al. found a decrease in serum IL-6 but not in serum IL-8 and TNF-alpha in ARDS patients through a reduction of Vt from 6 to 3 mL/kg of PBW achieved by extracorporeal CO_2_ removal [26]. Terragni et al. demonstrated a decrease in alveolar cytokines (IL-1beta, IL-1Ra, IL-6, and IL-8) after 72 h permitted by the reduction of Vt (6 to 4 mL/kg of PBW) during extracorporeal CO_2_ removal [27]. Interestingly, this was only observed in patients with 28 ≤ Pplat ≤ 30 at baseline but not in patients with lower Pplat (range 25–28 cmH_2_O) suggesting that benefit of reduction of Vt was more important in patients with evidence of tidal hyperinflation.

Thus, we hypothesize that the weak difference in Vt (≈ 1 mL/kg of PBW) and the same PEEP level (≈ 13 cmH_2_O), with baseline Pplat of 24–25 cmH_2_O between the ULP and LP ventilation strategies could explain the lack of differences between biomarkers in our study. Consequently, we found only minor differences in plateau pressure and no difference in driving pressure between groups. Median values of driving pressures were notably around 10 cm H_2_O whatever the group.

The impact of the respiratory rate by itself on biotrauma has not been largely studied. In our LP group, patients were ventilated ≈ 5 cycles more than in the ULP group during 48 h. In a ventilated rat model, Rich et al. [28] found no impact of high respiratory rate (40 vs 20 cycles/min) on biotrauma in animals ventilated with low Vt. However, impact of respiratory rate on VILI is more obvious above a certain degree of strain *per* respiratory cycle [29].

Our group has previously demonstrated a decrease in IL-1 beta in BAL after prone position in ARDS patients ventilated with Vt of 6 mL/kg/PBW (20). Contrarily, in the present study, we did not find differences in biotrauma when patients were placed in prone position for at least 16 h as compared with patients who remained in supine position. Importantly, all patients were ventilated with low Vt (< 4 mL/kg/PBW).

Finally, targeting a positive transpulmonary pressure may be associated with lower biotrauma in the experimental model of ARDS [30]. In quite a large randomized controlled study, Wang et al. confirmed the decrease in time course inflammatory cytokines after the application of a positive expiratory transpulmonary pressure-guided ventilation in patients on vv-ECMO as compared with a lung rest strategy [10]. This strategy was notably associated with lower mortality.

## Strengths and limitations

Despite the relatively small size of the study, there were no major baseline differences regarding clinical characteristics and biomarkers. There was no protocol deviation in each group, allowing some relevant differences regarding mechanical ventilation components. However, we agree that clinicians chose the lowest range of respiratory rate allowed in the LP group and the highest in the ULP group minimizing the differences in minute ventilation and mechanical power. Different results could also be expected from a protocol design targeting differences in driving pressures. Contrarily, the 48-h duration of the protocol would have been sufficient to demonstrate any difference. Also, only a minority of patients were switched to APRV mode in the LP group at 48 h which could have decreased the potential benefit of spontaneous breathing on biotrauma. Finally, we cannot also exclude a lack of power concerning 60-day and hospital mortality rates which almost reached statistical significance.

### Implications for future research

Determining the best way to set the ventilator during vv-ECMO needs additional clinical evidence and notably how far we can go to minimize lung stress and strain through ECMO while waiting for lung recovery. In the interim, the EOLIA ventilator protocol during ECMO is considered as a reasonable standard by experts [16]. Ultra-lung-protective ventilation with very low tidal volume may also enhance some additional risks such as the use of deep sedation and neuromuscular blockade to suppress any increase in respiratory drive or could promote atelectrauma resulting from the lower inspiratory airway pressures used. Although our ULP strategy was associated with minimized mechanical power, it is possible that an apneic ventilation would minimize VILI and biotrauma. Even if its feasibility has been demonstrated [31], this strategy is not widely adopted [10].

## Conclusion

A multimodal ultra-lung-protective strategy including intermittent prone position during 48 h in severe ARDS patients supported by vv-ECMO was not associated with a decrease in the pulmonary and the systemic biotrauma as compared with the lung-protective strategy of the EOLIA trial [17, 32]. Results of future or ongoing trials exploring clinical outcomes are expected [17, 32].

## Supplementary Information

**Additional file 1.** CONSORT 2010 checklist.

**Additional file 2.** Online data supplement.

## Data Availability

Individual patients’ data reported in this article will be shared after de-identification (text, tables, figures, and appendices), beginning 6 months, and ending 2 years after article publication, to researchers who provide a methodologically sound proposal and after approval of the first and last author.

## Abbreviations

APRV: Airway pressure release ventilation
ARDS: Acute respiratory distress syndrome
BAL: Broncho alveolar lavage
COVID-19: Coronavirus disease 2019
ECMO: Extracorporeal membrane oxygenation
LP: Lung-protective
PBW: Predicted body weight
PEEP: Positive end-expiratory pressure
P_*L*_: Transpulmonary pressure
PP: Prone position
Pplat: Plateau pressure
RR: Respiratory rate
ULP: Ultra-lung-protective
VILI: Ventilator-induced lung injuries
Vt: Tidal volume
vv-ECMO: Veno-venous membrane oxygenation
