# Supplementary material for: Ultra-lung-protective ventilation and biotrauma in severe ARDS patients on veno-venous extracorporeal membrane oxygenation: a randomized controlled study
Source: Crit Care. 2022 Dec 12;26:383. doi: 10.1186/s13054-022-04272-x (PMC9744058; doi:10.1186/s13054-022-04272-x)
Supplement: Supplementary file 2 — Additional file 2. Online data supplement. [file 13054_2022_4272_MOESM2_ESM.docx]

**ONLINE DATA SUPPLEMENT**

**SUPPLEMENTAL METHODS**

*Indication for vv-ECMO*

The decision to initiate ECMO was based on the following criteria: 1) persistent hypoxemia, defined as PaO_2_/FiO_2_ ≤ 70 mm Hg with FiO_2_ at 100 % and PEEP > 10 cm H_2_O for at least 2 hours, despite optimized mechanical ventilation (Vt set at 6 mL/kg PBW) and recourse to adjunctive therapies (neuromuscular blockers, prone positioning, inhaled nitric oxide) or 2) PaO_2_/FiO_2_ < 100 mm Hg with respiratory acidosis (pH <7.20) and Pplat > 35 cm H_2_O.

*Exclusion criteria*

Exclusion criteria were:

- va-ECMO support
- vv-ECMO support for more than 24 hours
- contra-indication for prone position
- contra-indication for esophageal pressure monitoring
- patients receiving steroids (> 0.5mg/kg/day of equivalent methylprednisolone)
- pregnancy
- patients deprived of liberty
- lack of social insurance

*Ethical statement and consent process*

The study was approved by the ethical committee “comité de protection des personnes Ouest VI” on Mai 23^th^, 2019. According to French law, written informed consent was obtained from the patients or their proxies either before study inclusion or, for patients not competent to provide consent and with no available proxies, at recovery of competence.

*Validity of esophageal pressure (P_es_) measurements, sedation management and respiratory mechanics formula used*

The adequate position of the balloon in the lower part of the esophagus was confirmed by presence of cardiac artifacts on the esophageal curve and a positive occlusion test (expiratory hold on the ventilator) in passive conditions with gentle chest compression (1). The occlusion test was considered as positive if the relationship between esophageal pressure and airway pressure should yield a slope of 1.0±0.2 cmH_2_O. At inclusion, all patients were deeply sedated to obtain a Richmond Agitation Sedation Scale (RASS) score of -5 and received continuous infusion of neuromuscular blockers to target a train-on-four of 0 on 4 twitches.

*Biomarkers list and assays details*

Biomarkers list and their specificity related to biotrauma are presented in **Table E.1**. They were chosen for their relevance in previous literature (2-4).

*Samples collection and processing*

For each patient, bronchoalveolar lavage (BAL) and arterial blood samples were collected in tubes containing ethylene diamine tetraacetic acid (EDTA) or without anticoagulant (Vacutainer, Becton–Dickinson, San Jose, CA) at inclusion and 48h after randomization. BAL and blood samples were rapidly centrifuged at room temperature, respectively at 300xg for 5 minutes and at 2500xg for 15 minutes. BAL supernatants, plasmas and serums obtained were aliquoted and stored at -80°C until analysis.

*Biomarker Assays*

Biomarkers were measured in BAL, plasma, or serum as indicated in **Table E.2** using commercially available ELISA and Luminex Assays kits : Angiopoietin-2, IL-1beta, sRAGE, TNF-R1 in BAL, VEGF-A, CC-16/uteroglobin, SP-D, MMP-9, IL-1Ra (Human Quantikine ELISA Kits, Bio-Techne, Minneapolis, MN, USA), Procollagen III (P3NP-ELISA, Cisbio, Codolet, France), vWF (TECHNOZYM vWF:Ag ELISA, Technoclone Herstellung von Diagnostika und Arzneimitteln GmbH, Vienna, Austria), Angiopoietin-4 (Human ANGPT-4 Elisa Kit, Abcam, Cambridge, United Kingdom), IL-6, IP-10 (Human Premixed Multi-Analyte Kits, Bio-Techne, Minneapolis, MN, USA), TNF-R1 in plasma, IL-8, IL-10, TNF alpha (Milliplex Kit, Merck Millipore, Burlington, MA, USA).

*Statistical analysis*

Data are expressed in median (interquartile ranges) or mean±sd according their distribution. Comparisons between groups are performed with the U Mann-Whitney-test or Student T-test as appropriate. For repeated variables, we used linear mixed-effects modeling fitting main effects for group, time, and an interaction between group and time. A *P* value less than 0.05 was considered as significant. All statistics and figures were performed with SPSS 20.0 software.

*Results*

Interim analysis was performed with PASS 15.0.11 after inclusion of 38 patients representing 63% of sample size. The distribution of alveolar interleukin-1 Beta in the experimental group was compared with the distribution of in the control group using the Mann-Whitney-test. A one-sided superiority test was conducted with a significance level on the total error of 0.20. Stopping boundaries were computed using a mean difference higher than 8±10 pg/mL in the control group. *P* value boundary >0.20 indicated termination of the study and acceptance of the null hypothesis (no difference of alveolar interleukin-1 Beta at H+48h between the experimental group i.e ULP group and the control group i.e LP group).

*Supplemental Figure Legends*

*Supplementary Figure S.1*

*Consolidated Standards of Reporting Trials diagram of the flow of patients through the study.*

ECMO= Extra Corporeal Membrane Oxygenation; ULP= Ultra-Lung-Protective; LP=Lung-Protective

*Supplementary Figure S.2*

*Respiratory mechanics and ventilator settings from cannulation to day 6*

Panel A: Tidal volume; Panel B: Respiratory rate; Panel C: Positive End Expiratory Pressure (PEEP); Panel D: Plateau pressure; Panel E: Driving pressure; Panel F: Mechanical power

*Supplementary Figure S.3*

*Panel A: Delta (T+48h minus T0) blood concentrations of tumor necrosis factor α (TNFα), Panel B: Delta (T+48h minus T0) blood concentrations of interleukin-8 (IL-8) and Panel C: Delta (T+48h minus T0) blood concentrations of vascular endothelial growth factor (VEGF)*

The empty circles represent the outliers.

**Supplementary Figure S.1. Consolidated Standards of Reporting Trials diagram of the flow of patients through the study**


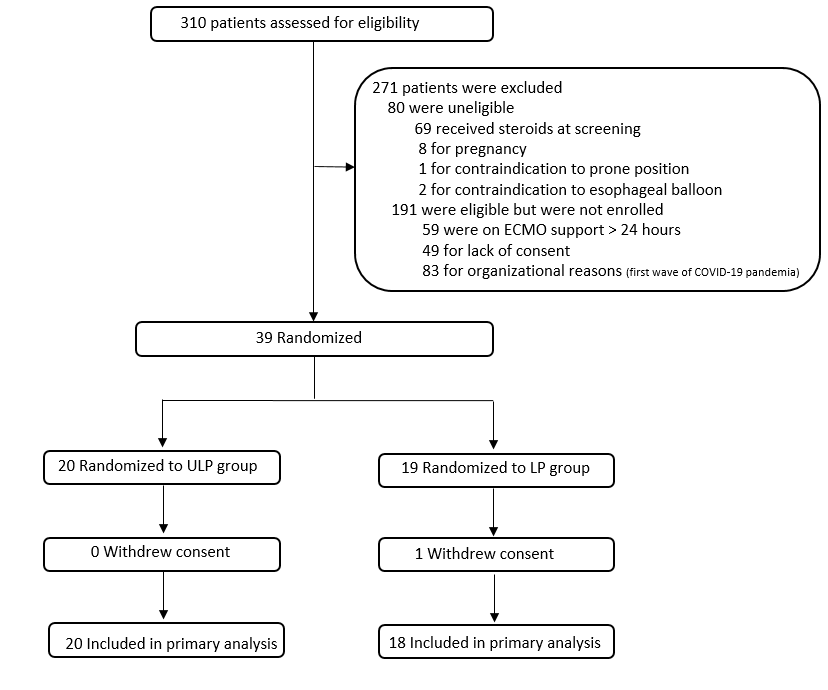


**Supplementary Figure S.2. Respiratory mechanics and ventilator settings from cannulation to day 6**


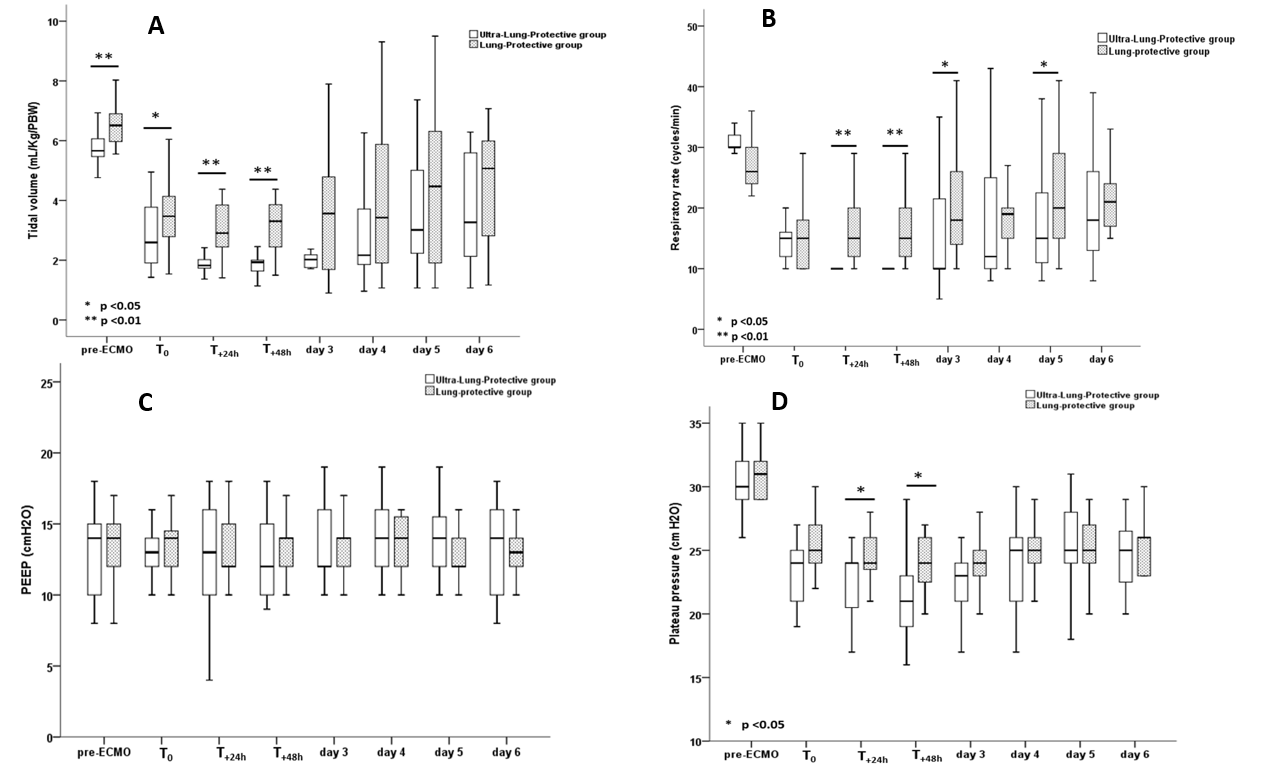


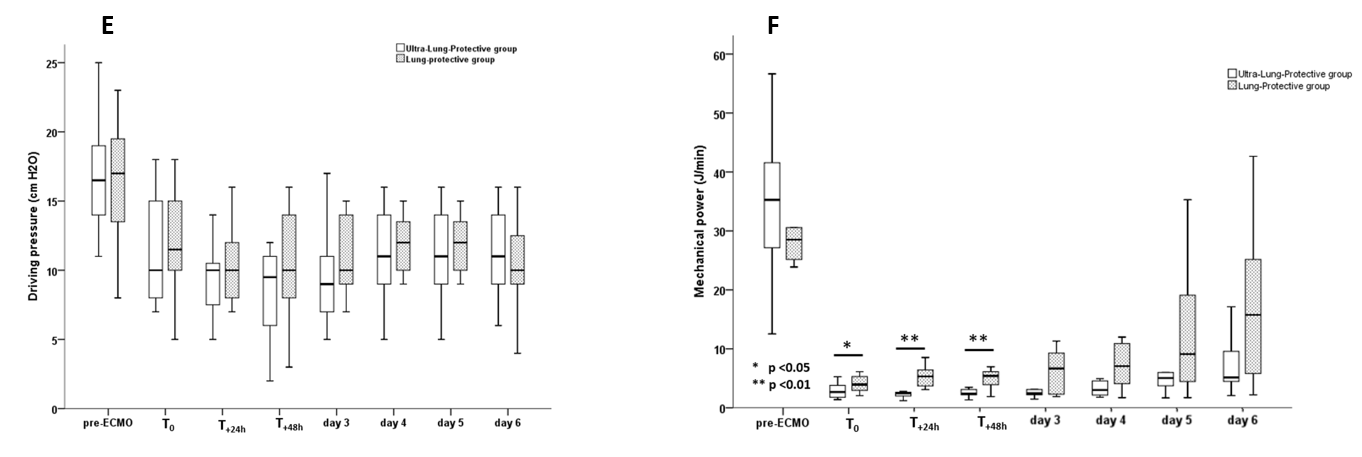


**Supplemental Figure S.3. Delta (T_+48h_ minus T_0_) blood concentrations of tumor necrosis factor α (TNFα), interleukin-8 (IL-8) and vascular endothelial growth factor (VEGF)**


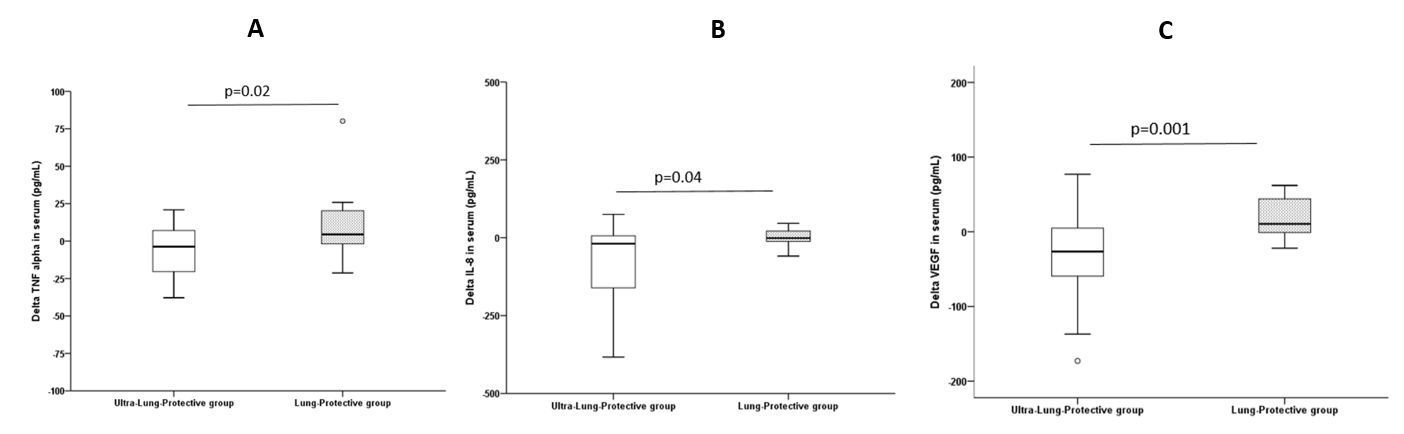


**Supplementary tables**

**Table S.1 Specificity and Sample site of each biomarker**


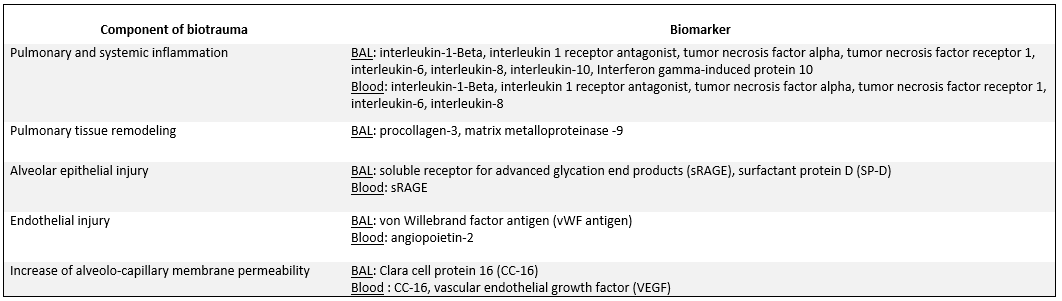


*Definition of abbreviations.* BAL= Broncho Alveolar Lavage

**Table S.2. Baseline concentrations of biomarkers**

| Biomarker | Primary outcome | Sample | Technic of measure, unit |  | Ultra-lung-protective group |  | Lung-protective group | *P* value |
| --- | --- | --- | --- | --- | --- | --- | --- | --- |
|  |  |  |  | *N* |  | *N* |  |  |
| Interleukin-1 Beta | Yes | BAL | ELISA, pg/mL | 20 | 40 (15-1140) | 18 | 79 (17-270) | 0.83 |
| Interleukin-1 Beta | No | Serum | ELISA, pg/mL | 20 | 0.45 (0.2-0.97) | 18 | 0.4 (0.2-0.9) | 0.78 |
| sRAGE | No | BAL | ELISA, pg/mL | 20 | 167 (57-5170) | 18 | 912 (50-20507) | 0.78 |
| sRAGE | Yes | Plasma | ELISA, pg/mL | 20 | 1045 (335-1402) | 18 | 565 (356-4203) | 0.76 |
| Angiopoietin 2 | Yes | Plasma | Luminex, pg/mL | 20 | 8642 (6576-14175) | 17 | 8168 (6306-18584) | 0.90 |
| TNF receptor 1 | No | BAL | ELISA, pg/mL | 20 | 951±499 | 18 | 741±472 | 0.19 |
| TNF receptor 1 | No | Plasma | ELISA, pg/mL | 20 | 5985±3598 | 18 | 6208±5341 | 0.88 |
| TNF alpha | No | BAL | Luminex, pg/mL | 20 | 6.9 (2.8-39.2) | 18 | 10.8 (1.4-59.5) | 0.94 |
| TNF alpha | No | Serum | Luminex, pg/mL | 20 | 27.5 (12.7-37.6) | 18 | 22.1 (10.9-34.4) | 0.42 |
| Interleukin-1ra | No | BAL | Luminex, pg/mL | 20 | 4.5 (1.4-9.5) | 17 | 4.5 (1.1-15.1) | 0.72 |
| Interleukin-6 | Yes | BAL | Luminex, pg/mL | 20 | 254 (57-1275) | 18 | 170 (100-415) | 0.90 |
| Interleukin-8 | No | Serum | Luminex, pg/mL | 20 | 50 (19-284) | 18 | 62 (24-92) | 0.83 |
| Interleukin-8 | Yes | BAL | Luminex, pg/mL | 20 | 512 (211-1265) | 18 | 710 (220-2155) | 0.57 |
| Interleukin-10 | No | Serum | Luminex, pg/mL | 20 | 30.4 (12.3-58.4) | 18 | 26.3 (10.2-72.8) | 0.42 |
| Interleukin-10 | No | BAL | Luminex, pg/mL | 20 | 7.3 (3.9-29) | 18 | 10.2 (2.7-76.2) | 0.96 |
| IP10 | No | BAL | Luminex, pg/mL | 20 | 253 (31-1021) | 18 | 211 (42-3062) | 0.83 |
| Procollagen 3 | No | BAL | ELISA, ng/mL | 20 | 3.6 (1.5-17.9) | 18 | 1.1 (1-5.3) | 0.08 |
| MMP-9 | No | BAL | ELISA, ng/mL | 20 | 252 (87-1516) | 18 | 348 (147-988) | 0.87 |
| Surfactant protein D | Yes | BAL | ELISA, ng/mL | 20 | 3 (0.4-33.8) | 18 | 3.8 (0.3-13.1) | 0.78 |
| Clara cell protein 16 | No | BAL | ELISA, ng/mL | 20 | 1462 (425-4892) | 18 | 1276 (337-5687) | 0.99 |
| Clara cell protein 16 | No | Plasma | ELISA, ng/mL | 20 | 58±38 | 18 | 61±39 | 0.87 |
| VEGF | No | Plasma | ELISA, pg/mL | 20 | 76 (47-157) | 18 | 48 (16-149) | 0.32 |
| vWF antigen | No | BAL | ELISA, mU/mL | 19 | 5.3 (1-41.8) | 18 | 4.3 (0.5-21.6) | 0.53 |

Values are expressed as median (interquartile range) or mean± standard deviation. *Definition of abbreviations and formula*: BAL=broncho-alveolar lavage; ELISA= enzyme-linked immune assay; sRAGE= serum advanced glycation end products; TNF=tumor necrosis factor; Interleukin-1ra= interleukin-1 receptor antagonist; IP10= Interferon gamma-induced protein 10; MMP-9=matrix metalloproteinase 9; VEGF= vascular endothelial growth factor; vWF= von Willebrand factor.

**Table S.3. Values of delta (T_+48h_ minus T_0_ ) biomarkers concentrations**

| Biomarker | Primary outcome | Sample | Technic of measure, unit |  | Ultra-lung-protective group |  | Lung-protective group | *P* value |
| --- | --- | --- | --- | --- | --- | --- | --- | --- |
|  |  |  |  | *N* |  | *N* |  |  |
| Interleukin-1 Beta | Yes | BAL | ELISA, pg/mL | 20 | -19 (-730 ; 21) | 18 | -8 (-212 ; 223) | 0.85 |
| Interleukin-1 Beta | No | Serum | ELISA, pg/mL | 19 | 0 (-0.3 ; 2) | 18 | 0 (-0.2 ; 0.3) | 0.99 |
| sRAGE | No | BAL | ELISA, pg/mL | 20 | -30 (-3758 ;172) | 18 | -10 (-2021 ; 420) | 1 |
| sRAGE | Yes | Plasma | ELISA, pg/mL | 20 | -151 (-745 ;14) | 18 | -106 (-463 ; 44) | 0.48 |
| Angiopoietin 2 | Yes | Plasma | Luminex, pg/mL | 20 | -284 (-5215 ; 127) | 17 | -12 (-2386 ; 3287 | 0.24 |
| TNF receptor 1 | No | BAL | ELISA, pg/mL | 20 | -87 (-491 ; 333) | 18 | 74 (-154 ; 207) | 0.34 |
| TNF receptor 1 | No | Plasma | ELISA, pg/mL | 20 | -288 (-1441 ; 1030) | 18 | 594 (-162 ; 1483) | 0.12 |
| TNF alpha | No | BAL | Luminex, pg/mL | 20 | -0.3 (-5.1 ; 5.4) | 18 | -0.4 (-18.5 ; 0.3) | 0.41 |
| TNF alpha | No | Serum | Luminex, pg/mL | 19 | -3.7 (-21.2 ; 7.3) | 18 | 4.4 (-1.8 ; 21.1) | 0.02 |
| Interleukin-1ra | No | BAL | Luminex, pg/mL | 20 | -0.1 (-3 ; 7.8) | 17 | -0.3 (-5.8 ; 1.63) | 0.62 |
| Interleukin-6 | Yes | BAL | Luminex, pg/mL | 20 | -24 (-219 ; 45) | 18 | 3 (-189 ; 139) | 0.63 |
| Interleukin-8 | No | Serum | Luminex, pg/mL | 19 | -24 (-83 ; 2) | 18 | -1 (-16 ; 31) | 0.04 |
| Interleukin-8 | Yes | BAL | Luminex, pg/mL | 20 | -41 (-231 ; 844) | 18 | -58 (-1712 ; 207) | 0.33 |
| Interleukin-10 | No | Serum | Luminex, pg/mL | 19 | -7.6 (-19.3 ; 5.2) | 18 | 0.1 (-11.5 ; 15.1) | 0.48 |
| Interleukin-10 | No | BAL | Luminex, pg/mL | 20 | -1.8 (-5.9 ; 15.3) | 18 | -0.5 (-14.9 ; 1) | 0.83 |
| IP10 | No | BAL | Luminex, pg/mL | 19 | 0 (-145 ; 213) | 18 | 20 (-285 ; 1009) | 0.60 |
| Procollagen 3 | No | BAL | ELISA, ng/mL | 20 | -0.25 (-4.7 ; 6.3) | 18 | 1.4 (0 ; 9.2) | 0.13 |
| MMP-9 | No | BAL | ELISA, ng/mL | 20 | -74 (-245 ; 48) | 18 | 103 (-138 ; 506) | 0.15 |
| Surfactant protein D | Yes | BAL | ELISA, ng/mL | 20 | 0.1 (-20.4 ; 3.8) | 18 | 5.3 (-1.6 ; 29) | 0.15 |
| Clara cell protein 16 | No | BAL | ELISA, ng/mL | 20 | 859 (-588 ; 5913) | 18 | 919 (-589 ; 2029) | 0.92 |
| Clara cell protein 16 | No | Plasma | ELISA, ng/mL | 20 | 0 (-12 ; 11) | 18 | -0.5 (-15 ; 11) | 0.38 |
| VEGF | No | Plasma | ELISA, pg/mL | 19 | -31 (-66 ; 5) | 18 | 10.5 (-1.2 ; 48.5) | 0.001 |
| vWF antigen | No | BAL | ELISA, mU/mL | 20 | -0.4 (-7.1 ; 4.6) | 18 | -0.3 (-3 ; 11) | 0.53 |

Values are expressed as median (interquartile range). *Definition of abbreviations and formula*: BAL=broncho-alveolar lavage; ELISA= enzyme-linked immune assay; sRAGE= serum advanced glycation end products; TNF=tumor necrosis factor; Interleukin-1ra= interleukin-1 receptor antagonist; IP10= Interferon gamma-induced protein 10; MMP-9=matrix metalloproteinase 9; VEGF= vascular endothelial growth factor; vWF= von Willebrand factor.

**Table S.4. Patients Outcomes**

| Outcomes | Ultraprotective ventilation | | Protective ventilation | | *P* value |
| --- | --- | --- | --- | --- | --- |
|  | *N* | *n (%)** | *N* | *n (%)** |  |
| Weaned off ECMO by day 60 | 20 | 12 (60) | 18 | 14 (78) | 0.24 |
| ECMO duration in survivors, days, median (IQR) | 20 | 10 (7-20) | 18 | 11 (8-23) | 0.56 |
| Weaned off ventilator by day 60 | 20 | 8 (40) | 18 | 9 (50) | 0.53 |
| 60-day mortality | 20 | 9 (45) | 18 | 3 (17) | 0.06 |
| ICU mortality | 20 | 8 (40) | 18 | 3 (17) | 0.11 |
| Hospital mortality | 20 | 9 (45) | 18 | 3 (17) | 0.06 |

*Definition of abbreviations*: ECMO=extracorporeal membrane oxygenation; ICU=intensive care unit. *Unless otherwise indicated.

**References**

1. Baydur A, Behrakis PK, Zin WA, Jaeger M, Milic-Emili J. A simple method for assessing the validity of the esophageal balloon technique. *Am Rev Respir Dis*. 1982; 126: 788-791. doi: 10.1164/arrd.1982.126.5.788. PMID: 7149443.
2. Ware LB, Calfee CS. Biomarkers of ARDS: what's new? *Intensive Care Med*. 2016; 42:797-799.
3. Terpstra ML, Aman J, van Nieuw Amerongen GP, Groeneveld AB. Plasma biomarkers for acute respiratory distress syndrome: a systematic review and meta-analysis. *Crit Care Med*. 2014; 42: 691-700.
4. Van der Zee P, Rietdijk W, Somhorst P, Endeman H, Gommers D. A systematic review of biomarkers multivariately associated with acute respiratory distress syndrome development and mortality. *Crit Care*. 2020; 24: 243. doi: 10.1186/s13054-020-02913-7. PMID: 32448370; PMCID: PMC7245629.
